# On making a difference: towards a minimally non-trivial version of the identity of indiscernibles

**DOI:** 10.1007/s11098-021-01647-8

**Published:** 2021-04-30

**Authors:** David Wörner

**Affiliations:** Department of Philosophy, Universität Zürich, Grünauring 20, 8064 Zurich, Switzerland

**Keywords:** Metaphysics, Identity, Identity of indiscernibles, Grounding

## Abstract

The identity of indiscernibles (PII) states that indiscernible objects must be identical. Many philosophers have held that the PII turns out to be either true but trivial, or non-trivial but false, depending on how the notion of (in)discernibility is spelled out. In this paper, I propose and defend an account of this notion which aims to yield a minimally non-trivial and yet plausible version of the PII. I argue moreover that this version of the principle is immune to a number of well-known and recent objections to the PII.

## Introduction

The principle of the identity of indiscernibles, or PII for short, states that, necessarily, indiscernible things are identical. The PII is and has been extremely controversial. Critics of the principle typically try to refute it by pointing out actual or possible cases of distinct but, allegedly, *in*discernible objects.^1^ Friends of the PII have responded in two main ways: either they have disputed that the objects the PII’s critic claims to be distinct genuinely are distinct,^2^ or they have argued that the objects in question, though distinct, turn out to be *discernible* after all.^3^ This last strategy, on which I shall focus in what follows, brings to the fore the question of what it *means* for things to be discernible: if we claim that certain distinct objects really are discernible even though they may seem not to be, we had better specify some rather robust account of what exactly discernibility is. But what is to guide our investigation into the notion of discernibility?

Some proponents of the PII think of it as a *consequence* of some metaphysical backstory they are independently committed to. If you think, for example, that an individual is nothing over and above a bundle of shareable properties, then you seem to be committed to the view that distinct things cannot share all their properties, and so, in a sense, cannot be “indiscernible.”^4^ If we approach the PII in such a way, our backstory will largely determine the notion of discernibility at issue in our version of the PII. This is not, however, the approach of the present paper. For the PII can also be treated as a *self-standing* principle, a principle that deserves attention in its own right. On such an approach, the main requirement the notion of discernibility has to meet is that it not render the PII *trivial*. If we say that things are not only distinct, but discernible, we imply that they satisfy some demand over and above the fact that they are distinct. If, moreover, we want to examine a version of the PII that has the best chance of being true, we want this demand to be as modest as possible: we want the demand to yield a *minimally non-trivial* version of the PII.

In this paper I propose an account of how to spell out this demand. My guiding thought is, roughly, that a minimally non-trivial version of the PII requires that there be a difference between all distinct objects and that this difference not hold between them *in virtue of* their distinctness. In Sects. 2–4, I introduce and motivate this proposal, and argue that it is superior to the account of a minimally non-trivial PII offered by Gonzalo Rodriguez-Pereyra (2006). The main payoff of my proposal is that a version of the PII becomes available which is more robust than often supposed. In particular, as I argue in Sect. 5, Max Black’s famous case of a symmetric universe and some more recently leveled objections by Katherine Hawley (2009) and Steven French (2019) fail to refute the principle if the notion of discernibility is understood along the lines I propose.

I should note at the outset that the account I put forward relies on a notion of *metaphysical ground*. I understand this notion broadly along the lines of Kit Fine’s (2012) account. In particular, I assume throughout the paper that grounding is factive, transitive and irreflexive, and that there is a useful distinction between full and partial ground. I take grounding to be a relation that holds between facts, even though nothing substantial depends on this.^5^ I mainly use the “in virtue of”-locution and the connective “because” to express grounding.

Before I begin, a brief note on how I understand the notion of identity. On the present approach, identity is considered to be a reflexive relation satisfying Leibniz’s Law. That is, it is a relation characterized by the following principles:

$$({\text{Ref}})\quad \square \forall x\forall y\left( {x = x} \right)$$

$$({\text{LL}})\quad \square \forall x\forall y\left( {x = y \to \forall F\left( {Fx \leftrightarrow Fy} \right)} \right).$$

This entails that identity is an equivalence relation. As instanced in my formulation of (LL), I shall adopt the framework of modal second order predicate logic throughout this paper.^6^

## Paradigmatic and non-paradigmatic differences

In its most general form, the PII purports to be a necessary truth connecting identity to a notion of discernibility: necessarily, things that are indiscernible are the same. Some philosophers understand discernibility in epistemic terms and consequently argue that “discernible” things are things *we can discern.*^7^ This is not how I shall understand the notion in what follows. Rather, I take the PII to be a strictly *metaphysical* principle, according to which for things to be “discernible” is for them to *differ*.^8^ To avoid misunderstanding, the following discussion focuses on the notion of a difference rather than that of discernibility: the PII is the principle that, necessarily, things that do not differ are the same. The PII, in its most general shape, may thus be put in more formal terms as follows, where “Diff” expresses the relation of differing:

$$({\text{PII}})\quad \square \forall x\forall y\left( {\neg {\text{Diff}}\left( {x,y} \right) \to x = y} \right)$$

The central question I want to address is what it takes for distinct things to differ in a way that renders the PII non-trivial, but yet plausibly true.

When we ordinarily think of *differences* between objects, we typically think of purely qualitative differences, such as differences in color, shape, volume, material or other such familiar features. It is not hard to say how a red ball differs from a blue cube—they differ by having different colors and shapes. We may think of such differences as *paradigmatically non-trivial* differences. If all objects necessarily differed in these ways, there would be no question that the PII is non-trivially true. Imagine a world in which all objects have different colors. Clearly, the PII, as restricted to that world, takes note of a substantive and informative feature of the world in question.

However, as most famously Max Black (1952) has argued, a version of the PII that requires distinct things to differ in these paradigmatically non-trivial ways surely is false. Black envisions a universe containing nothing but two iron spheres at some distance from each other. The spheres are perfectly alike with regard to their color, material, shape, and so on. There are *no* paradigmatically non-trivial differences between them. Yet such a universe is possible.^9^ So the proponent of the PII has to look for differences between the spheres that *fail* to be paradigmatic differences.

To be sure, it is not hard to find *some* relations between the spheres that, granted some leniency in our use of the term, may be called “differences.” For instance, dubbing one sphere *Castor* and the other *Pollux*, we may say that the spheres differ with regard to the properties of *being Castor*, *being Pollux*, *not being Castor*, and *not being Pollux.* These “differences,” call them *haecceitic* differences, are non-paradigmatic, and one may rightly insist that they are indeed *too far* from being paradigmatic to help the PII’s case. If the PII is true merely because it is a necessary truth that all things have their own unique haecceitic property of being the very things they are, then surely the PII is less than what its proponents have wanted it to be. Similar words apply to other types of difference. For example, Black’s spheres differ from each other because each of them is a member of a unique singleton. Castor is a member of {Castor}, Pollux is not. Still, it is trivially true that all distinct things must belong to different extensionally defined sets. Such differences, again, are too far from the paradigmatic ones to help the PII’s case. So the challenge the friend of the PII has to meet in responding to cases such as Black’s universe is one of specifying the sort of difference that has to hold between all distinct objects in a way that *neither* restricts those differences to the paradigmatic ones *nor* allows them to trivialize the PII. Call differences which would trivialize the PII *trivial* differences, for short, and call the differences a non-trivial version of the PII requires to hold between distinct objects *substantial* differences.

A first response to Black’s case on part of the proponent of the PII may be to include differences that consist in bearing a *relation* to something among the substantial differences. The paradigmatic differences listed above are differences in non-relational, intrinsic properties. Apart from those, things may differ with regard to how they are related to other objects. A red ball and a blue cube, for instance, may differ because only one of them sits on my table, or because only one of them has the same color as a cloudless sky. Suppose that a third object apart from the spheres, a golden cube, say, occupies Black’s universe. Assume further that the spheres are at differing distances to the cube. We may then say that the spheres differ substantially since only one of them, say, Castor, is one mile apart from a golden cube. Castor bears the relation of being one mile from a golden cube, whereas Pollux bears the complement of that relation to a golden cube. Even though this difference may not count as a paradigmatic difference, it is not a trivial truth that the spheres differ in this way. If it were necessarily true that any two objects bear incompatible relations, there would be a substantial sense in which we could say that any two objects must differ.

However, Black’s universe is set up to contain *nothing* but the two iron spheres. They do not bear any relation to any object apart from themselves. If they differ with regard to the relations they bear, these relations must be the ones that hold between the spheres themselves. But then, it seems that they share most if not all the relations they stand in. Each sphere bears, for example, the relation of being of the same color, volume, shape, and so on, as an iron sphere. Furthermore, assuming that the spheres are two miles apart from each other, each sphere bears the relation of *being at a distance of two miles from an iron sphere.* These relations cannot constitute differences between the spheres—all of them are *symmetric* over the pair of them.

Looking for a relation borne by one but not the other sphere, we may take recourse to so called “impure” properties: to extrinsic properties consisting in relations to *one particular individual*. Castor, for example, bears the property of *being two miles from Pollux*, and Pollux lacks this property. But does this difference make the spheres different in the sense demanded by a version of the PII that is still non-trivial? Is it a trivial truth (if it is a truth at all) that all distinct objects differ with regard to impure properties? This is a controversial question. Hawley (2009) and others have recently argued that such differences presuppose (in a sense yet to be specified) the distinctness of the objects between which they hold.^10^Rodriguez-Pereyra (2006), by contrast, has proposed a version of the PII which allows for objects to differ merely with regard to their impure properties but is yet (he claims) non-trivial and substantive. To adjudicate between these positions, we need a criterion by reference to which we can decide what sorts of differences trivialize the PII.

Differences in impure properties are not the only type of difference whose status as substantial differences is contested. Taking up a suggestion by Quine (1976), we need not appeal to impure properties to specify *some* way in which Black’s spheres may be said to “differ” from each other. For each sphere is two miles from the other sphere, but none of them is two miles apart *from itself.* The relation of being two miles apart, in other words, is symmetric but *irreflexive* over the pair of them. Things bearing symmetric but irreflexive relations may, in accordance with current philosophical lingo, be called *weakly* discernible. The claim that all distinct things are discernible in at least this sense—the so called *weak discernibility proposal—*has garnered a lot of attention in the philosophy of quantum physics. For contrary to a long-held consensus, Simon Saunders and others have argued that even the existence of presumably indiscernible quantum particles, such as entangled fermions and bosons, does not refute the PII—if, that is, the PII is construed along the lines of the weak discernibility proposal.^11^ For even such particles, they argue, bear symmetric but irreflexive relations to each other. Part of the criticism that has been leveled against this proposal concerns technical issues particular to the philosophy of quantum physics.^12^ But other parts concern the very notion of weak discernibility itself. In particular, as we shall see in more detail below, Steven French (2019) has argued that appealing to irreflexive relations always presupposes, in some sense, the distinctness of the things to be discerned, and that the weak discernibility proposal therefore fails to articulate a non-trivial version of the PII. Again, this calls for an investigation into what it takes for the PII to be non-trivial, and what a minimally non-trivial version of the principle entails.

Summing up, then, there are two important types of non-paradigmatic difference which may or may not count as substantial differences: differences in impure properties, call them *impure* differences, and differences consisting in symmetric but irreflexive relations, call them *weak* differences. The challenge for the PII’s proponents can now be specified more precisely. It is to draw a line between different sorts of non-paradigmatic difference: to keep out clearly trivial differences, such as haecceitic differences or differences in membership to extensionally defined sets, on the one hand, but yet to let in at least some impure or weak differences. If such a line can indeed be drawn, then there is at least a good chance for a—minimally non-trivial—version of the PII to emerge that is able to withstand the most well-known scenarios that have been put forward against it.

## Rodriguez-Pereyra on trivializing properties

A good starting point for distinguishing trivial from substantial differences is the thought that things that differ non-trivially are not *merely* distinct.^13^ Accordingly, one way of putting the point of the PII is to say that things cannot differ *solo numero.* A substantial difference is one that is something over and above numerical distinctness. The tricky thing is to specify more precisely what this means—precisely enough, that is, to decide whether impure and weak differences may be substantial or not.

The most sustained attempt at meeting this challenge is due to Rodriguez-Pereyra (2006). Rodriguez-Pereyra’s approach is premised on a certain account of the logical form of the PII: for him, the PII is a principle that states that things are identical if there is no *property* with regard to which they differ. Thus, any version of the PII takes the following form^14^:

$$({\text{PII}}^{\prime } )\quad \square \forall x\forall y\left( {\forall F\left( {Fx \leftrightarrow Fy} \right) \to x = y} \right)$$

This account of the PII’s logical form commits Rodriguez-Pereyra to a certain conception of the logical form of the notion of a *difference*: any difference is a difference *in property*. The most general notion of a difference may thus be defined as follows:

$$({\text{Diff}})\quad {\text{Diff}}\left( {x,y} \right) =_{{{\text{def}}.}} \exists F\left( {Fx \wedge \neg Fy} \right).$$

Given this framework, defining a notion of a substantial difference is a matter of restricting the property domain of the second order quantifier figuring in (Diff): differences with regard to certain properties are substantial, while differences with regard to others are trivial. Rodriguez-Pereyra frames his discussion in terms of an attempt to define the latter: the properties, that is, that must be excluded from the property domain in a definition of a substantial difference. He calls these properties *trivializing properties*, as any version of the PII which includes them in its property domain is trivially true. His proposed definition of the notion of trivializing property runs as follows^15^:

(RP): *F* is a trivializing property = _def._ Differing with respect to *F* is or may be differing numerically.^16^

Rodriguez-Pereyra’s explanation of what he has in mind when he writes that differing with regard to a certain property “is or may be” differing numerically is rather sparse. The best way of approaching his meaning is to consider his account of why certain properties count as trivializing according to (RP). He holds that differing with regard to haecceitic properties and their complements *is* differing numerically: for Socrates to differ from Plato with regard to the property of *being Socrates* or that of *not being Plato* just is for them to be distinct. Apart from such properties, all trivializing properties are trivializing because differing with regard to them “may be” differing numerically. Take the conjunctive property of *being Socrates and being a person.* According to Rodriguez-Pereyra, differing with regard to this property “may be” differing numerically. This is so, he writes, because “[a] conjunctive property is such that having or lacking it is having or lacking other properties. So differing with respect to *F&G* is simply differing with respect to *F* or *G* or both. So differing with respect to *F&G* may consist in differing with respect to *F*” (Rodriguez-Pereyra, 2006, 216). Very similar words apply, on his account, to disjunctive properties such as that of *being Socrates or being green*: since having that property may be having the property of *being Socrates*, differing with regard to it may be differing with regard to that property and thus differing numerically (ibid.).

There are some properties that seem problematic for Rodriguez-Pereyra’s account. Consider the trivializing property of *being a member of singleton {Socrates}.* As Rodriguez-Pereyra (2006, 217) acknowledges, it is at least *prima facie* unclear why this property should qualify as trivializing on his account. For it does not seem obvious that differing with regard to singleton membership “may be” differing numerically. In order to resolve this apparent problem, Rodriguez-Pereyra proposes an account of singleton membership on which (RP) qualifies them as trivializing. “[T]he property of *being a member of {a},”* he suggests, *“*is the property of *being such that {a} exists and being identical to a*” (ibid.)*.* Given such an account, the property of being a member of {Socrates} turns out to be trivializing on (RP). Both Socrates and Plato are such that {Socrates} exists, but only Socrates is identical to this singleton’s member. Their difference with regard to membership to {Socrates} accordingly consists in nothing but their difference with regard to being Socrates.

There are, however, other trivializing properties that cause trouble for (RP) even if we grant Rodriguez-Pereyra’s account of singleton membership. For Socrates not only has the property of *being such that singleton {Socrates} exists*; he also has the property of *grounding the existence of singleton {Socrates}*.^17^ Surely, the fact that each individual grounds the existence of a unique singleton cannot be enough to establish a substantive, non-trivial version of the PII. The property of grounding a given singleton’s existence is a trivializing property. But it is hard to see why it is trivializing according to (RP). It is implausible to hold, say, that for Socrates to ground the existence of {Socrates} *just is* for him to be Socrates. For Socrates also grounds the existence of singletons that do not contain him as a member: by grounding the existence of {Socrates}, Socrates also grounds the existence of the singleton containing the singleton {Socrates}, given that grounding is transitive. Yet, of course, for Socrates to ground the existence of the singleton containing {Socrates} is not for him to *be* the singleton {Socrates}. At least *prima facie,* therefore, (RP) fails to cover all trivializing properties.

## Trivial differences as partially grounded in distinctness

Rodriguez-Pereyra (2006, 217) explicitly contrasts his notion of actually or possibly *consisting* in a numerically difference to the notion of holding *in virtue of* a numerical difference. What makes the property of *being Socrates and being a person* trivializing, on his account, is not that Socrates and Plato differ with regard to this property *in virtue* of being distinct—it is that for them to differ with regard to the property *is* for them to be distinct.^18^ I think that this contrast lies at the heart of what is wrong with (RP). For once we do think of trivializing properties in terms grounding, it becomes possible to accommodate all trivializing properties in a straightforward way.

Consider the following alternative to (RP), an account which I shall call *distinctness dependence*, or (DD) for short:

(DD): *F* is a trivializing property with regard to *x* and *y* =_def._ *x* and *y* differ with regard to *F* partly in virtue of the fact that *x* is distinct from *y.*

Note that (DD), in contrast to Rodriguez-Pereyra’s account, defines a trivializing property in relation to a given pair of objects. A property may be trivializing with regard to one pair of objects but not with regard to another. Take the property of *being Socrates or being green*. Socrates differs merely trivially from Plato with regard to this property, as Plato lacks the property in virtue of not being Socrates. A red ball, however, differs *non-trivially* from a green cube with regard to the property in question, as the difference between those objects does not hold in virtue of their distinctness.^19^

All of the most obvious examples of trivializing properties are trivializing according to (DD): Socrates and Plato differ with regard to the property of *being Socrates and being human* and that of *being Socrates or being green* partly in virtue of being distinct. If we assume, moreover, that in general things *have* (or lack) a property *F* in virtue of being (or not being) *F*, we can also easily see why the property of being distinct from Plato qualifies as trivializing on (DD)^20^: Socrates and Plato differ with regard to the property of being Socrates in virtue of being distinct, as Plato lacks that property in virtue of not being Socrates.^21^

Importantly, (DD) also captures the trivializing properties that are problematic for Rodriguez-Pereyra’s account. Consider first properties of singleton membership. It is not hard to admit that Plato lacks the property of being a member of {Socrates} in virtue of *not* being Socrates. Thus, the property is trivializing according to (DD). Second, take properties of grounding the existence of singletons. Socrates and Plato in that Socrates, but not Plato, has the property of grounding the existence of {Socrates}. If we hold—plausibly—that Plato lacks this property in virtue of not being Socrates, the property turns out to be trivializing as well, given (DD). We do not need to reinterpret these properties in terms of haecceitic properties in order to qualify them as trivializing if we assume (DD). Rodriguez-Pereyra’s account of singleton membership may be correct, and it may even be possible to construe properties of grounding the existence of singletons in terms of haecceities. Even so, the advantage of (DD) is that it, in contrast to (RP), does not *commit* us to such accounts.

Rodriguez-Pereyra prefers (RP) to an approach along the lines of (DD) because he thinks that the latter mistakenly qualifies properties as trivializing which should *not* be so qualified. Here is his argument:

But what if it were the case that all of the properties of a thing were had in virtue of being *that* thing? In that case *a* would have all of its properties simply in virtue of being *a*. Perhaps the world is like that. Perhaps things cannot share all their properties because they have their properties in virtue of being the things they are. Or perhaps things cannot share all their properties because every thing has a qualitative property that is necessarily peculiar to it in virtue of being the thing it is. In either case PII would be true but it would be non-trivially true. For even if things were qualitatively different in virtue of being numerically different, differing qualitatively would still be more than differing numerically. (Rodriguez-Pereyra, 2006, 217)

Consider first the claim that something may have a unique “qualitative property” in virtue of being the very thing it is: let us assume that Socrates, in virtue of being Socrates, has a unique “Socratesness” that is qualitative *in some sense*. Even so, it does not follow that Plato *lacks* “Socratesness” in virtue of *not* being Socrates: it does not follow that Socrates differs from Plato with regard to “Socratesness” partly in virtue of the fact that he is distinct from Plato. For it may be that Plato has some other property—his “Platoness,” perhaps—in virtue of which he does not possess “Socratesness.” A scarlet ball differs from an azure one with regard to the property of *being scarlet.* This does not entail that the azure ball lacks that property in virtue of *not being scarlet*—rather, the azure ball lacks the property in question in virtue of *being azure.* Similar considerations apply to a world in which things have *all* their properties in virtue of being the very things they are. That Socrates has the property *F* in virtue of being Socrates, and that Plato, who is distinct from Socrates, lacks *F*, does not entail that Plato lacks *F* in virtue of being *distinct* from Socrates. But then, both possibilities Rodriguez-Pereyra raises are consistent with a non-trivial version of the PII that is spelled out along the lines of (DD).

One might think that there are other non-trivializing properties which (DD)—wrongly—counts as trivializing. Consider the property of *loving someone other than oneself*. Assume that the nymph Echo loves Narcissus, who only loves himself. Echo and Narcissus accordingly differ with regard to the property in question. Then, (DD) counts the property as trivializing with regard to the pair of Narcissus and Echo. For, presumably, Echo’s possession of the property of *loving someone other than oneself* is grounded in the two facts that she loves Narcissus and that she is distinct from Narcissus. One might think that this result is problematic for (DD): for does it not seem that Narcissus and Echo differ substantially? After all, Narcissus emerges from the present scenario as a narcissist, while Echo does not.

Let me point out in reply that the present case involves other differences between Narcissus and Echo, differences over and above their difference with regard to the property of *loving someone other than oneself*. We have assumed, for example, that Narcissus, loving himself only, does not love Echo. Therefore, Echo lacks the property of being loved by Narcissus, while Narcissus has that property. This property is *not* trivializing on (DD), as surely neither the fact that Echo is not loved by Narcissus nor the fact that Narcissus is holds in virtue of the fact that Echo is not Narcissus. Moreover, this difference seems metaphysically prior to the difference regarding the property of *loving someone other than oneself*: plausibly, the fact that Narcissus only loves himself is partially grounded in the fact that he does not love Echo. So our reluctance to count the difference with regard to *loving someone other than oneself* as a trivial difference may issue from the fact that this difference holds partly in virtue of another difference which *is* substantial. Accordingly, one might suggest slightly to revise (DD) such that differences that are partially grounded in substantial differences count as differences regarding a non-trivializing property, even if they are partially grounded in a numerical difference. It seems to me, however, that (DD) captures the distinction between trivial and non-trivial differences well enough and that the suggested revision, though available, is not needed. For (DD) suffices to show that, if Narcissus and Echo differ with regard to *loving someone other than oneself*, they differ substantially with regard to at least one property—and this means that they differ in way that is consistent with a minimally non-trivial version of the PII.

With these clarifications about the notions of trivializing properties and trivial differences in place, let me now return to the most well-known alleged counterexample to the PII. If the possibility of Black’s universe is to refute a minimally non-trivial version of the PII, then the two spheres the universe contains must differ *merely* trivially. As I shall argue now, there is at least a plausible case to be made that this is not the case.

## Do Black’s spheres differ non-trivially?

Let me begin with the question of whether Black’s spheres differ with regard to a non-trivializing *impure* property. The spheres, call them again *Castor* and *Pollux*, differ with regard to the impure properties of *being at a distance from Castor* and of *being at a distance from Pollux.* Are these properties trivializing?

Katherine Hawley (2009) has put forward an argument to the effect that such differences are grounded in the distinctness of the things between which they hold—and so are trivial in the sense articulated by (DD).^22^ She focuses on the impure properties of *being two miles from a* and of *being two miles from b.* She argues (2009, 109–110) in detail, and I think convincingly, that “the distinctness of the two monadic properties is grounded in the distinctness of the two objects, *a* and *b*” (2009, 110). She then takes for granted that this result entails that the *difference* between *a* and *b* regarding these properties is also grounded in the fact that *a* and *b* are distinct, immediately inferring that her argument shows that “we cannot take the fact that *a* and *b* are discernible in respect of these properties to ground the fact that *a* and *b* are distinct” (ibid.). Thus, Hawley’s argument is premised on the implicit assumption that whatever grounds the distinctness of the *properties* with regard to which two things differ also grounds the *difference* in question. However, it is not at all obvious that this assumption is correct.

The difference between the spheres—call them, again, *Castor* and *Pollux*—regarding the impure properties of *being two miles from Castor* and *being two miles from Pollux* holds in virtue of four facts:

(1a): Castor has the property of being two miles from Pollux
(1b): Pollux lacks the property of being two miles from Pollux
(1c): Pollux has the property of being two miles from Castor
(1d): Castor lacks the property of being two miles from Castor

Since grounding is transitive, whatever grounds these four facts also grounds the difference between the spheres regarding the two properties in question. And, presumably, each of these facts is fully grounded in its respective counterpart among the following facts^23^:

(2a): Castor is two miles from Pollux
(2b): Pollux is not two miles from Pollux
(2c): Pollux is two miles from Castor
(2d): Castor is not two miles from Castor

What about the fact that the two impure properties at issue are *distinct*? So far, this fact does not enter our explanation of why the spheres differ with regard to the properties in question at all. It is one thing to specify what grounds the *possession* (or lack) of a property; it is quite another thing to specify what grounds the *distinctness* of properties possessed by different objects. Hawley’s argument, it seems to me, precipitately equates these matters.

If there is a case to be made for the claim that facts (1a)–(1d) are partially grounded in the spheres’ distinctness, it has to be based on the claim that facts (2a)–(2d) are. Note that these latter facts are just the ones a proponent of the weak discernibility proposal would allude to in order to discern Black’s spheres: for to specify these facts is to specify a symmetric but irreflexive relation—that of *being two miles apart*—which the spheres bear to each other. So given that (1a)–(1d) hold in virtue of (2a)–(2d), we may say that the spheres differ impurely *because* they differ weakly. The next question to address, therefore, is whether their weak difference is trivial or not.

I have so far assumed that the PII takes the logical form specified in (PII’) and that the notion of a difference to be defined is a notion of a difference *in property* along the lines of (Diff). Once we take weak differences into account, however, this approach is inadequate. A weak difference holds between two objects if and only if they bear a symmetric but irreflexive relation to each other; and such differences are not differences in property. To accommodate weak differences, we need a more general account of the logical form differences take. The most obvious candidate for such an account defines a difference in terms of disjunction, in which the second order variable “*R*” ranges over relations^24^:

$${\text{(Diff}}^{\prime } {)}\quad {\text{Diff}}^{\prime } (x,y) =_{{{\text{def}}.}} \exists F\left( {Fx \wedge \neg Fy} \right) \vee \exists R\left( {Rxy \wedge \neg {\text{Rxx}}} \right).$$

If we plug in (Diff’) for (Diff) in (PII), the resulting version of the PII is the one put forward by proponents of the weak discernibility proposal:

$$({\text{PII}}^{\prime \prime } )\quad \square \forall x\forall y\left( {\neg \left( {\exists F\left( {Fx \wedge \neg Fy} \right) \vee \exists R\left( {Rxy \wedge \neg {\text{R}}xx} \right)} \right) \to x = y} \right).$$

In order to specify a minimally non-trivial version of this principle, we need an account, not only of trivializing properties, but of trivializing *relations*: relations that are such that, if they are included in the domain of the quantifier ranging over relations in (PII’’), render the resulting version of the PII trivial. In keeping with the general idea that trivial differences are partially grounded in distinctness, let me propose the following account, which is intended to complement (DD):

(DD′): A relation *R* that holds between *x* and *y* is trivializing =_def._ either (i) *R* is distinctness itself, or (ii) *R* holds between *x* and *y* partly *in virtue of* the fact that *x* and *y* are distinct.

With this account in mind, let me approach some of the main worries that have been brought forward regarding the weak discernibility proposal.

French, for one, has argued that the weak discernibility proposal is unsuited as a version of the PII for very general, principled reasons. After having discussed a number of technical difficulties with the weak discernibility proposal as applied to quantum particles, he writes that

there is the further philosophical concern that the appeal to irreflexive relations in order to ground the individuality of the objects which bear such relations involves a circularity: in order to appeal to such relations, one has had to already individuate the particles which are so related and the numerical diversity of the particles has been presupposed by the relation which hence cannot account for it. (French, 2019, 13)

French’s point is couched in epistemic terms, even though he clearly intends to make a metaphysical point: he means that the individuation of objects must, in some sense, be metaphysically prior to their differing weakly.

As far as I can see, there are two ways in which one may try to substantiate French’s reasoning in the above quote. One of them concerns the fact that weak differences are *relations* that cannot be captured in terms of differences in properties. French and Krause (2006, 172) justify their claim that weak differences fail to “ground individuality” by referring to a thesis of Ruth Barcan Marcus’s: “Individuals,” Barcan writes, “must be there before they enter into relations”.^25^ In terms of metaphysical ground, Barcan’s claim is most plausibly read to entail that neither the *existence* nor the *identity* of individuals is grounded in any part in their bearing relations to each other. Perhaps, the claim is moreover intended to imply that individuals stand in relations partly in virtue of being the very individuals they are. I do not want to challenge these claims here.^26^ For these claims anyway do not bear on the present, minimally non-trivial version of the PII. This version of the PII involves no ambition at all to ground the existence or identity of individuals. Accordingly, Barcan’s thesis is quite consistent with the possibility that distinct things may differ non-trivially in the sense at issue merely by bearing symmetric but irreflexive relations. If Barcan’s thesis is to bear on the present version of the PII, it must entail that “individuation” is prior to bearing relations in the sense that individuals bear all relations apart from distinctness itself partly *in virtue of being distinct*. But this claim is much stronger—and less intuitively appealing—than the claim that individuals exist and are the very individuals they are prior to bearing relations to each other. We need some reason to believe that, say, Castor is two miles apart from Pollux partly in virtue of being distinct from Pollux—and the general idea that individuals are in some sense prior to bearing relations does not provide such a reason.

A second way of justifying French’s reasoning in the passage quoted above may be based on a consideration recently offered by Erica Shumener (2017). Shumener attempts to show that the weak discernibility proposal cannot be appealed to in order to ground distinctness, and her argument appears to be relevant in the present context as well. As Shumener (2017, 4) reconstructs it, the claim that two individuals *a* and *b* are distinct because they differ weakly is the claim that the fact that *a* is distinct from *b* is grounded in two facts:

(3): *Rab*
(4): *R* is irreflexive

Shumener (ibid., 4–5) goes on to argue that this claim is viciously circular because the fact that *R* is irreflexive is itself grounded in the fact that its relata are distinct. If this argument were sound, it would equally refute the idea that weak differences may be non-trivial in the sense of (DD’), as it would establish that weak differences are partly grounded in the distinctness of the things between which they hold.

But Shumener’s argument is based on a misleading and ungenerous reading of the position she criticizes. For given *a* and *b*, the weak discernibility proposal is not that *a* and *b* differ weakly in virtue of facts (3) and (4). For *a* and *b* to differ weakly with regard to *R* is for each of them to bear *R* to the other but not to itself. This fact holds in virtue of the following facts:

(3): *Rab*
(5): $$\neg Raa$$
(6): *Rba*
(7): $$\neg Rbb$$

The fact, say, that Castor differs weakly from Pollux is *not* grounded in the facts that the relation of *being two miles apart* holds between them and that the relation of *being two miles apart* is irreflexive. Rather, Castor and Pollux differ weakly because in virtue of the facts (2a)–(2d): in virtue of the facts that Castor is two miles from Pollux but not from itself and Pollux is two miles from Castor but not from itself. These facts of course entail that the relation of *being two miles apart* is irreflexive over the pair of the spheres. But the relation’s irreflexivity does not *itself* enter the ground of the fact that the spheres differ weakly. The proponent of the weak discernibility proposal need not rely on any fact as to which relations are irreflexive; she need only rely on facts about which objects bear, or fail to bear, relations to each other or themselves.

Summing up, the two ways in which French’s worry about the weak discernibility proposal may be vindicated both ultimately fail to put pressure on a minimally non-trivial version of the proposal. So far, then, we have not encountered any general and principled reason to think that weak differences must be trivial. There is nothing about the notion of a weak difference that prohibits weak differences from being substantial, non-trivial differences.

## Conclusion

I have aimed in this paper to articulate a minimally non-trivial version of the PII and to examine, in general terms, what sorts of differences this principle requires to hold between distinct things. A minimally non-trivial PII is one that is true if and only if, necessarily, all distinct things differ non-trivially. I have argued that the notion of a trivial difference at issue is best captured in terms of *grounding*: a trivial difference is one that either consists in the numerical diversity of its relata or is partially grounded in their numerical diversity. On the basis of this account, I have defended the claim that some cases of non-paradigmatic differences—impure and weak differences—may be non-trivial. The minimally non-trivial version of the PII that emerges from the present discussion is thus weaker than is often supposed—and accordingly harder to refute.

To be sure, the previous considerations do not show, and do not aim to show, that the minimally non-trivial version of the PII I have specified is *true*. Perhaps there are actual or possible cases, particularly in the domain of quantum particles, of distinct things that differ merely trivially. What I have hoped to pin down, however, is what exactly such cases need to involve: they need to involve distinct objects that differ only in respects which are partially grounded in their very distinctness. This conception helps get out of the way a number of worries that are based on more ambitious versions of the PII. And it establishes a ground rule by reference to which objections to the PII may be assessed in a principled way.

## Footnotes

1 The most well-known putative counterexample, which also takes center stage in the following discussion, is that put forward by Black (1952). For a rather comprehensive list of such putative counterexamples, see Muller (2015, 203–204).

2 Hawthorne (1995) outlines one way of pursuing this strategy; see also Hawthorne and Sider (2002); for criticism see Hawley (2009). Moreover, Hacking (1975) has argued that all cases that putatively contain distinct but indiscernible objects can be described in such a way that the putatively indiscernible objects are identical; see Adams (1975) for criticism. Della Rocca (2005) has put forward an argument that Black’s universe is impossibe; see again Hawley (2009) for criticism.

3 Recent proponents of this strategy most notably include advocates of the “weak discernibility proposal.” I discuss the weak discernibility proposal below (Sects. 2 and 5).

4 Note, however, that some recent authors have argued that bundle theories can be dissociated from the claim that things sharing all properties must be identical; see Rodriguez-Pereyra (2004) and Demirli (2010).

5 Some philosophers who are sympathetic to using the notion of ground wish to main neutral on whether there is a grounding *relation* (see Correia 2010; Fine 2012) and so prefer expressing it exclusively by way of sentential connectives instead of predicates.

6 However, none of the arguments presented depend on this choice, as the second order principles may be rephrased as first order schemata. Such a reformulation, however, renders the notion of discernibility relative to a language—and I do not want to presuppose this here.

7 For instance, discussing putative counterexamples to the PII, Ladyman and Bigaj write that “we should make sure that there is a proper physical procedure that could in principle identify those objects as distinct from the remainder of the universe that does not discern them” (2010, 128) and they go on to argue that the PII requires that the differences holding between distinct objects “can really be said to allow the particles to be ‘discerned’” (ibid., 131).

8 To be sure, this way of putting things is not without its own difficulty. Sometimes “to differ” and “to be different” are used synonymously with “to be distinct.” This, of course, is not the sense of the terms I have in mind here.

9 This, at least, is what I shall assume in what follows, even though some proponents of the PII hold that Black’s universe is impossible if it is to contain two distinct spheres. Della Rocca (2005) has argued that we should consider the universe to be impossible because, if we do not, we lack any reason to prohibit objects from being completely colocated. I think that Hawley (2009, 103–104) has successfully shown this argument to be flawed.

10 Black himself holds that predicates expressing impure properties cannot be alluded to articulate a difference between the spheres because such predicates would be constructed by using proper names uniquely referring to the spheres. Black thinks that there cannot be proper names uniquely designating the spheres of his universe (see Black 1952, 156–157). I do not share Black’s worries about proper names; still, I shall not discuss them in any detail; note that even if Black is correct and we cannot refer to the spheres by using proper names, we can articulate “weak” differences (as explained below) that hold between the spheres.

11 French (1989) provides a classic statement of the view that cases of entangled quantum particles refute the PII. The weak discernibility proposal is championed, among others, by Saunders (2006), Muller and Saunders (2008), Muller and Seevinck (2009) and Muller (2015).

12 See French and Krause (2006), ch. 4.2.1, Hawley (2009), Ladyman and Bigaj (2010) and Bigaj (2015) for examples of such criticism. I should note that I am absolutely not in a position to competently take a stance on the more technical issues regarding the weak discernibility proposal in quantum physics.

13 Alternatively, one might begin with the idea that the PII requires distinct things to differ “qualitatively.” Note, however, that it is not obvious that all non-qualitative differences are trivial differences: perhaps a non-trivial version of the PII is true even though there are some distinct things that differ merely in ways that are not qualitative, in *some* sense of the term.

14 The notation used here slightly differs from Rodriguez-Pereyra’s. Moreover, he does not formulate the PII as stating a necessary truth. For the purposes of this paper, these differences are irrelevant. I follow Rodriguez-Pereyra in treating all versions of the PII as involving second order quantification. There are other ways of formulating various versions of the PII. Most commonly, the principles are considered to be first order schemata, and the notion of a difference is consequently defined relative to a language. Using second order quantification facilitates the following discussion, although none of my claims and arguments depend on this choice, as far as I can see.

15 Rodriguez-Pereyra begins his discussion by rejecting two attempts at defining the notion of a trivializing property, one of which runs in terms of property entailment and the other in terms of property containment. Roughly, the first approach attempts to specify trivializing properties on the basis of the idea that trivializing properties are those which “entail” haecceitic properties; the second approach [which is based on the approach taken by Katz (1983)] characterizes trivializing properties as ones that “contain” haecceitic properties. As I think that Rodriguez-Pereyra’s refutation of these accounts succeeds, I shall not discuss it in any detail.

16 Except for the name Rodriguez-Pereyra gives to his proposal, this is a direct quote from Rodriguez Pereyra (2006, 219).

17 This formulation is somewhat imprecise. For saying that Socrates has the property of grounding the existence of singleton {Socrates} suggests that grounding is a relation between an *individual—*Socrates—and a fact—the fact that singleton {Socrates} exists. However, along with many authors working on the topic, I shall assume that grounding is first and foremost a relation between *facts*, not between individuals and facts. More precisely, then, the property in question here is not that of grounding the existence of singleton {Socrates}, but that of *being such that one’s existence grounds the existence of singleton {Socrates}.* The following considerations can be rephrased in this more verbose way.

18 Talk of what a property “consists in” may be more intimately related to the notion of grounding than Rodriguez-Pereyra assumes, see Fabrice Correia and Skiles (2019). Alex Steinberg (2019), moreover, has recently argued that “just is”-statement to the effect that for some fact A to hold *just is* for some fact B to hold can be analysed in terms of grounding. In the present paper, I shall assume for the sake of argument that Rodriguez-Pereyra is right in thinking that for a property or fact A to consist in some property or fact B prohibits A from grounding B.

19 One and the same property may even be trivializing with regard to a given pair of objects even though it is not trivializing with regard to a third object and one member of the pair: the property of *being Socrates or being green*, for instance, is not a trivializing property with regard to Plato and a green cube.

20 For a more detailed discussion of this assumption, see Fine (2012, 67–71). I should note that Rodriguez-Pereyra appears to assume that the assumption is false. On his account, for some object to have the property *F just is* for that object to be *F*. And as we have seen, he refuses to spell out this notion in terms of grounding.

21 As an anonymous reviewer has pointed out, the proposed account does not capture certain properties involving higher order quantification which do render the PII trivial if anything instantiates them. Consider the property of being *indiscernible with regard to any binary relation*: $F_{R} (x) =_{{{\text{def}}.}} \forall R\left( {\forall y\forall z\left( {Ryx \leftrightarrow Rzx} \right) \wedge \forall y\forall z\left( {Rxy \leftrightarrow Rxz} \right)} \right).$ Suppose that *a* and *b* both instantiate this property. This logically entails that *a* and *b* are identical. Restricted to this case, the PII will thus be trivially true. But note that if any object instantiates *F*_*R*_, it follows that it is the *only* object there is. Any version of the PII is trivially true in a world which contains only one object. In order to keep the PII from being trivially true in worlds which contain distinct objects, therefore, it is not necessary to exclude properties such as *F*_*R*_ from its property domain. On the present account, then, such properties are not trivializing properties.

22 To be sure, Hawley herself presents the argument as an objection to the weak discernibility proposal, even though it is much more pertinent to the idea that impure properties of the sort at issue here may be alluded to discern objects.

23 I again assume a view broadly along the lines of Fine’s (2012, 67–71) take on the relationship between grounding and property abstractions.

24 This definition may also be simplified if we assume that any difference in property entails a difference that consists in the bearing of an irreflexive symmetric relation. This assumption is plausible given that any two things that differ with regard to a property *F* bear the symmetric and irreflexive relation of *differing with regard to F.*

25 French and Krause take this latter quote from Ruth Barcan Marcus (1993, 200).

26 To be sure, they may have to be modified in order to accommodate, for example, the idea that some individuals may ontologically depend on their parts and the relations that hold between those parts. Furthermore, Muller (2015) has recently argued that objects that differ merely by bearing symmetric irreflexive relations belong to a special category of “relationals.” Note, however, that Black’s spheres, for example, surely belong to the same ontological category as the middle-sized dry goods of our ordinary experience. Imagine that one of Black’s spheres would suddenly contain a scratch on its surface; then there would be more than a mere weak difference between the spheres. But should we really say that it would make the spheres *change their category*? I do not think that we should bring into play such metaphysical machinery in order to defend the weak discernibility proposal.
